# Comprehensive Analysis of Laser Peening Forming Effects on 5083 Aluminum Alloy

**DOI:** 10.3390/mi15080949

**Published:** 2024-07-24

**Authors:** Chuijiang Kong, Xiaojun Zhang, Gongling Chen, Xiamin Yuan, Bing Liu, Ran Zhu

**Affiliations:** 1 College of Mechanical and Electronic Engineering, Shandong University of Science and Technology, Qingdao 266590, China; kongchuijiang@163.com (C.K.); sdkdzxj@163.com (X.Z.); yxm2965345@163.com (X.Y.); metrc@sdust.edu.cn (B.L.); 2 Caoxian Science and Technology Innovation Service Center, Heze 274400, China; chengl6@163.com; 3 Key Laboratory of Urban Rail Transit Intelligent Operation and Maintenance Technology & Equipment of Zhejiang Province, Jinhua 321004, China

**Keywords:** laser peening forming, deformation, aluminum alloy skin, 5083 aluminum alloy

## Abstract

In order to investigate the laws of the laser peening forming process and the effects of laser peening on the surface quality and tensile properties of 5083 aluminum alloy, experiments were conducted utilizing various laser peening paths, energies, and plate thicknesses. Subsequently, laser peening forming experiments were performed on S-shaped and different shapes of aluminum alloy substrates. The impact of different laser peening durations on surface morphology and tensile properties was then analyzed. Results indicated that the largest bending deformation perpendicular to the laser peening path reached 12.5 mm. In cases where the laser peening path was inclined relative to the horizontal direction, torsional deformations were observed in the aluminum alloy plate. For laser energy levels of 5 J, 6 J, and 7 J, deformation amounts were 3.8 mm, 4.9 mm, and 5.4 mm, respectively. Plates with thicknesses of 4 mm exhibited convex deformation, while those with 2 mm thickness showed concave deformation. Furthermore, following one and two laser peening cycles, the residual stresses in the alloy plates were −80 MPa and −107 MPa, the surface hardness increased by 16 HV and 31 HV, the roughness increased by 2.495 μm and 3.615 μm, and the tensile strength increased by 9.5 MPa and 18.5 MPa, respectively.

## 1. Introduction

Alloy 5083 aluminum is extensively utilized in automotive, aircraft, and marine industries for its high strength-to-weight ratio, excellent resistance, and welding capabilities [1,2,3]. The growing demand for metal parts in electronic products and microelectromechanical systems (MEMS) has drawn interest towards forming technologies associated with them. Nevertheless, conventional forming methods are characterized by high costs, lengthy cycle times, and limited precision [4].

In recent years, laser-shock-forming technology has emerged as a novel approach to address the aforementioned issues. Laser Shock Form (LSF) is a process used to shape plates by generating high strain-rate plastic deformation through the application of a laser shock wave [5]. Hackel et al. [6] introduced the concept and methodology for plastic deformation of plates through laser peening. Hammersley et al. [7] proposed a laser peening forming technique that relies on pre-bending, where the plate undergoes initial elastic bending before being shaped through laser peening. This approach significantly enhances the level of plastic deformation in the plate. Ocana et al. [8] studied the deformation of a cantilever beam made of stainless-steel plate girder with a laser peening of 50 um thickness. They discovered that the overall deformation was a combination of the local concave surface at the focal point and the macroscopic bending deformation of the plate girder. Sagisaka et al. [9] conducted a laser peening forming method on SUS304 stainless steel to experiment with a large-angle precise convex bending process in order to obtain various shapes of bent parts. Pence et al. [10] conducted a study on the bending deformation mechanism and analyzed the influence of various process parameters on workpiece forming. Nagarajan et al. [11] conducted experiments in the United States to determine the maximum bending angle and forming efficiency of laser-shot peening under different process parameters. They also investigated the impact of laser-shot peening on material properties. Mao et al. [12] conducted laser peening on a 12.7 mm thick steel plate to induce the formation of micron-sized microstructures on its surface. This process aimed to enhance the friction properties of the steel plate. Zhou et al. [13] conducted a theoretical analysis of the plate-deformation process and developed a theoretical model for the deformation of a single plate subjected to laser peening. Hu et al. [14] investigated the laser-peening forming process concerning the direction of laser incidence, distinguishing between convex and concave bending directions. They proposed a stress-gradient mechanism and an impact-bending mechanism, noting the coupling effect between the two mechanisms. Yang et al. [15] conducted a study on the laser peening forming of 7075 alloy sheets with thicknesses of 0.6 mm and 3 mm. They proposed a laser peening forming system based on a specific strategy. In a separate study, Rao al. [16] performed experiments on the deformation of 2024 aluminum alloy through laser peening. They analyzed the impact of various process parameters, such as laser energy, impact area, spot overlap rate, and specimen thickness, on the deformation of the alloy. The results indicated that larger laser pulse energy and smaller target material thickness led to concave deformation in the specimen, whereas smaller energy and larger target material thickness resulted in convex deformation. Wang et al. [17] selected 2024-T351 aerospace aluminum alloy thin-walled parts as the subject of their study. They conducted experimental research on the laser peening strengthening method with bilateral asynchrony. It was observed that employing bilateral asynchronous laser peening on both sides of the target material yields a uniform residual stress field. Moreover, the macro-deformation remains within the required precision range of the shape. Ding et al. [18] used a combined numerical and experimental approach to determine that plate thickness is a critical factor affecting laser peening forming. Jian et al. [19] developed a process-based spreading technique that relies on specific deformation behaviors to determine the initial blank shape for LPF (Laser Peening Forming). Their findings demonstrate that the process-based method yields a more precise initial blank than the geometry-based approach, ultimately enhancing the accuracy of the forming. Although a large number of studies have been carried out on laser-peening forming, most of them focus on the influence of the relevant impact parameters on forming and investigate the mechanism of laser-impact forming. There are fewer studies on the comprehensive analysis of laser-peening forming and the properties of the formed plates.

This study focuses on investigating the process of laser peening on 5083 aluminum alloy plates. We adopted a systematic approach to test various laser peening paths, energy levels, and plate thicknesses. The effects of laser peening on the surface quality and tensile properties of the aluminum alloy were analyzed. Additionally, experiments were conducted to evaluate the laser peening process on S-shaped parts and aluminum alloy substrates of different shapes.

## 2. Materials and Methods

### 2.1. Experimental Material

5083 cold-rolled aluminum sheets were used in this study, primarily composed of aluminum with minor quantities of magnesium, manganese, chromium, and other trace elements. The chemical composition, expressed in weight percentage (wt%), is as follows: 95.2% Al, 3.5% Mg, 0.5% Mn, 0.3% Si, 0.26% Cr, and 0.24% Fe [20]. Annealing was carried out to remove internal stresses before conducting the experiments. The surface of the specimen was ground and polished to remove the oxide film on the surface of the specimen, and greasy dirt was removed using acetone.

### 2.2. LSP Experiments

**Figure 1:**
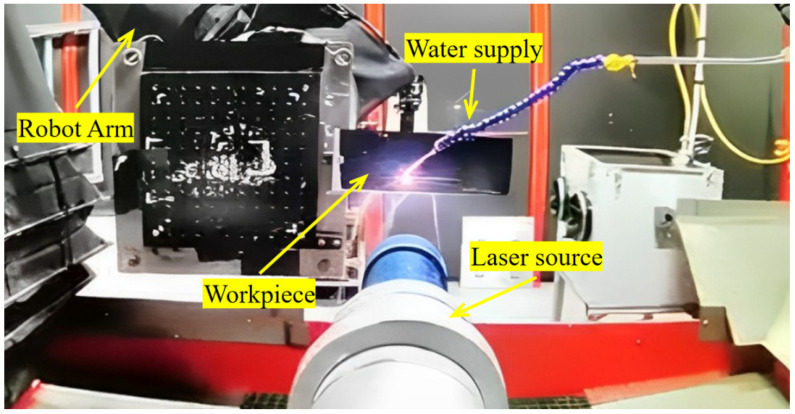
PROCUDO200 laser blasting system.

As shown in Figure 1, a third-generation laser blasting system (LSPT, Dublin, OH, USA, PROCUDO200) was used for the test. The key technical parameters were as follows: all laser rods were YLF crystals, pump diode lifetime than 15 years, pulse energy 5 J–10 J, pulse maximum repetition frequency 20 Hz, pulse width 8–16 ns, spot size adjustable between 2 mm and 8 mm, and laser wavelength 1053 nm.

### 2.3. Experimental Study of Laser Peening Forming Process Rules

**Figure 2:**
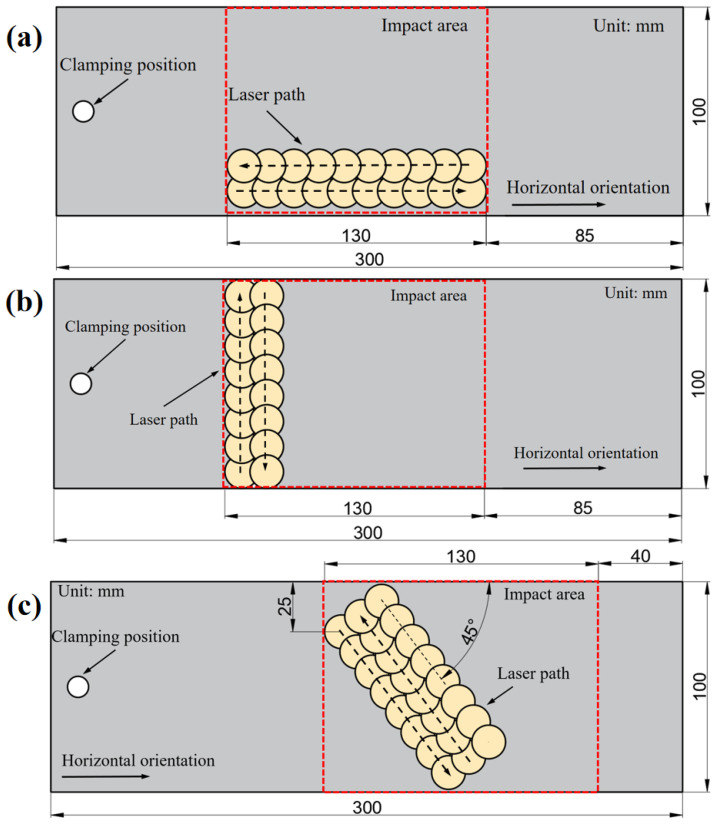
Laser peening paths: (**a**) Impact path 1: parallel to horizontal; (**b**) Impact path 2: perpendicular to horizontal; (**c**) Impact path 3: inclined to horizontal.

To study the law of laser peening forming on 5083 aluminum alloy sheets, we chose a 300 mm × 100 mm × 4 mm sheet of this alloy as our research object. Figure 2 illustrates the three different laser-peening paths that were designed. To explore their impact on the forming trend of the aluminum alloy plate, we used a unilateral-bolt fixed constraint with an impact area of 130 mm × 100 mm. Next, we selected the laser-peening path parallel to the horizontal direction and conducted forming tests on 5083 aluminum alloy plates of varying thicknesses: 2 mm, 4 mm, and 5 mm. This allowed us to analyze the influence of different thicknesses on the bending deformation of the plates. Finally, we focused on the 4 mm thick 5083 aluminum alloy plate and performed laser-peening tests using laser energy levels of 5 J, 6 J, and 7 J. Our goal was to examine the effect of different laser-energy levels on the bending deformation of the aluminum alloy plate.

**Figure 3:**
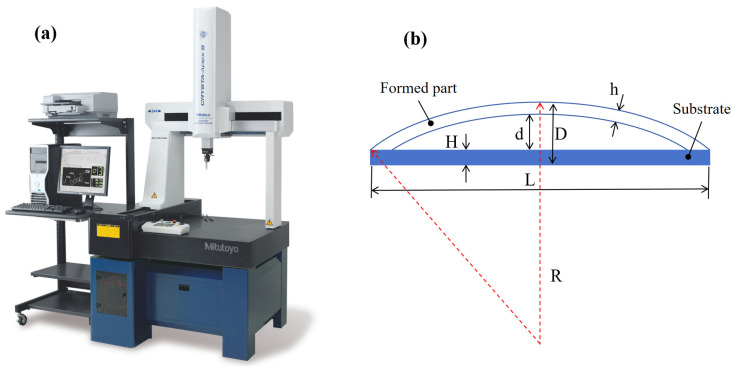
Measuring instruments and measuring methods; (**a**) Coordinate measuring machine; (**b**) arc bow height schematic.

To measure the degree of deformation of the plate, we considered the following dimensions: the thickness of the aluminum alloy plate is h, the thickness of the substrate is H, the length of the substrate is L (300 mm), the radius of curvature of the bent plate is R, the arc height for bending the plate is d, and the maximum distance between the bent plate and the substrate is D. We used the arc height to gauge the extent of deformation in the plate post-bending. In order to obtain the arc bow height, a coordinate measuring machine (CMM) was used to measure the coordinates of the plate after deformation and calculate the arc bow height. The CMM is shown in Figure 3a. The schematic diagram of the arc bow height is shown in Figure 3b.

### 2.4. Effect of Laser Peening on Surface Quality and Tensile Properties of Aluminum Alloy Materials

A plate with a size of 300 mm × 100 mm × 4 mm was used for double-sided laser peening treatment. The laser pulse frequency was set at 5 Hz, with a spot size of 3 mm and a flat-top distribution beam. The remaining process parameters are specified in Table 1. X-ray testing was used to measure the residual stress of the aluminum alloy plate after laser-peening treatment. Specific measurements were made by the side-dipping fixed Ψ method, with the fixed-peak method being the mutual correlation method; the radiation was CuKα and the diffracted crystal plane (213). Microhardness and surface roughness were observed using a microhardness tester and a white light interferometer, respectively. Additionally, the surface morphology of the specimen was examined.

**Figure 4:**
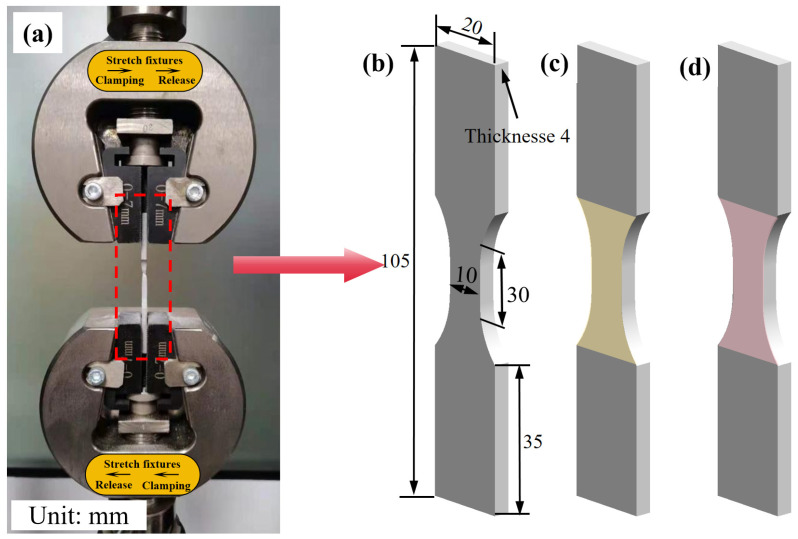
Dimensions of laser-shock treated tensile specimens; (**a**) Electronic universal testing machine (WDW-200); (**b**) Zero laser shock treatment; (**c**) Laser shock treatment once; (**d**) Laser shock treatment twice.

The aluminum alloy plate was used to create the tensile specimen pieces, which were cut using wire cutting. The dimensions of these specimens are illustrated in Figure 4b. The tensile experiment was conducted utilizing an electronic universal testing machine (Bairoe, Shanghai, China, WDW-200), depicted in Figure 4a. The tensile speed was set at 2 mm/min, with a gauge length of 30 mm for the tensile specimen. Following tensile fracture, the fracture morphology was observed using a TM3030 benchtop microscope(Hitachi High-Tech, Schaumburg, IL, USA, TM3030).

### 2.5. Laser Peening Forming of Aluminum Alloy Plates for S-Shaped Profiles

**Figure 5:**
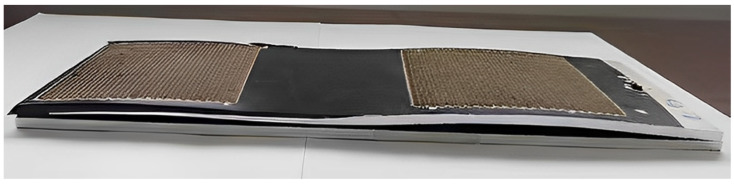
Results of laser peening molding of S-shaped shaped parts.

A laser with an energy of 5 J, a pulse width of 16 ns, and a frequency of 5 Hz, focused on a 3 mm spot, was applied to the aluminum alloy plate; the resulting S-shaped laser-peening effect is shown in Figure 5. The measured arc bow height is 5.1 mm.

### 2.6. Laser Peening Forming of Aluminum Alloys with Different-Shaped Substrates

**Figure 6:**
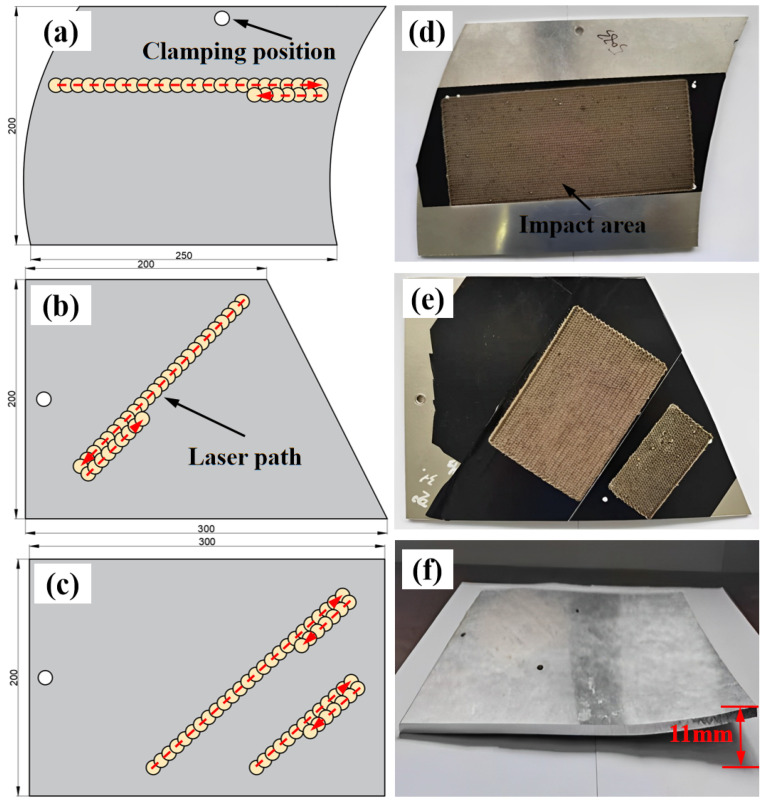
Effect of LSP forming on different shapes of substrates. (**a**) Geometry of a C-shaped plate; (**b**) Geometry of a T-shaped plate; (**c**) Geometry of a Square-shaped plate; (**d**) Results of LSP on C-plate; (**e**) Results of LSP on T-plate; (**f**) Results of LSP on Square-plate.

The same laser-peening parameters were used to perform tests on plates with different initial shapes, as shown in Figure 6a–c. The results of the laser peening forming are presented in Figure 6d–f. As illustrated in Figure 6d, the aluminum alloy substrate predominantly underwent bending deformation, whereas in Figure 6e,f, the substrate primarily underwent torsional deformation. The maximum displacement of the highest point of the torsion-formed plate with respect to the horizontal plane was 11 mm.

## 3. Results and Discussion

### 3.1. Influence Law and Analysis of Process Parameters

#### 3.1.1. Effect of Laser Peening Path on the Bending Deformation of Aluminum Alloy Plates and Its Analysis

**Figure 7:**
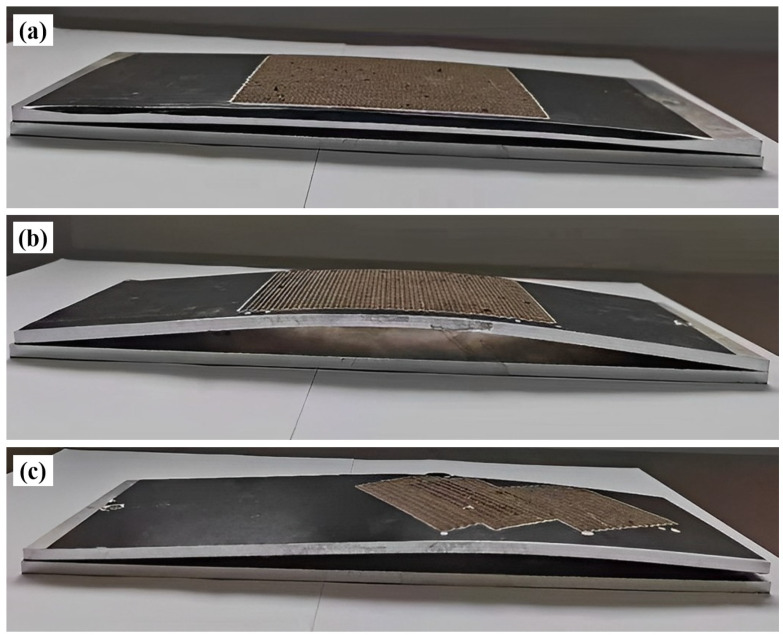
Effect of different laser peening paths on plate forming: (**a**) Impact path 1; (**b**) Impact path 2; (**c**) Impact path 3.

**Figure 8:**
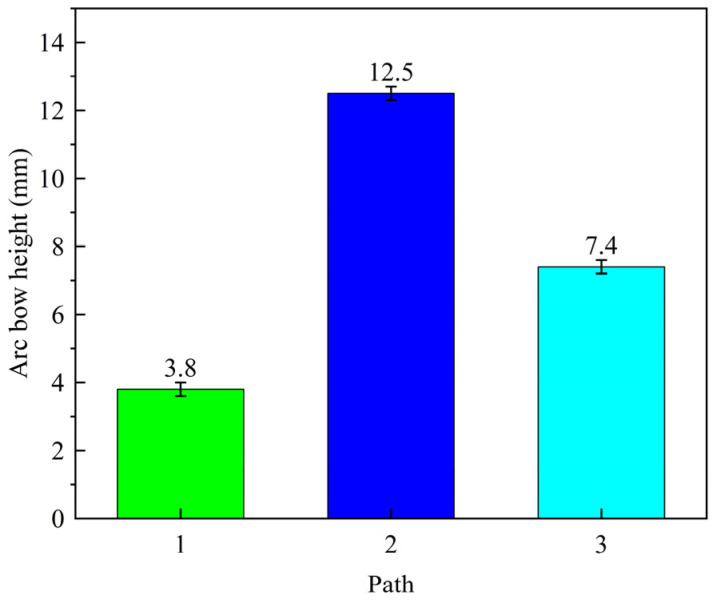
Effect of laser peening path on arc bow height.

For the laser peening forming experiments on 300 mm × 100 mm × 4 mm 5083 aluminum alloy plates, a laser energy of 5 J, a pulse width of 16 ns, a frequency of 5 Hz, and a spot size of 3 mm were selected. The effects of different laser peening paths are shown in Figure 7, and the impact of the laser peening path on the height of the arc bow is illustrated in Figure 8.

**Figure 9:**
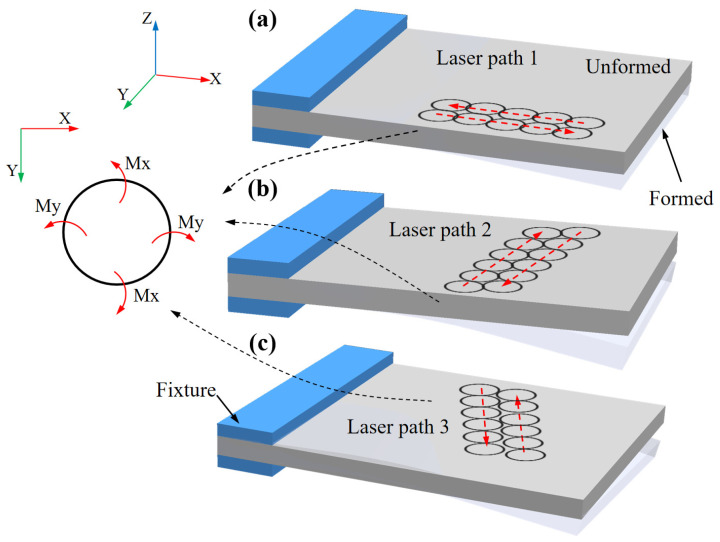
Schematic diagram of plate bending moment synthesis under different impact paths: (**a**) Laser path 1; (**b**) Laser path 2; (**c**) Laser path 3.

As shown in Figure 7 and Figure 8, under the same laser process parameters, a 4 mm aluminum alloy plate exhibited convex deformation relative to the direction of laser incidence. Shock path 2 resulted in greater deformation of the plate, with an arc height of 12.5 mm, compared to impact paths 1 and 3. This difference is primarily due to the formation of residual stresses and bending moments around the *X* and *Y* axes in the region of a single small spot. In Figure 9a, when laser peening was carried out along path 1, the impact path was parallel to the *X*-axis (the length direction of the plate), resulting in opposite bending moments around the *Y*-axis between neighboring spots, which reduced the bending deformation around the *Y*-axis. Conversely, in Figure 9b, when laser peening was performed along path 2, the bending moments around the *Y*-axis between neighboring spots were in the same direction, thereby enhancing the bending deformation around the *Y*-axis [14]. Comparing impact paths 1 and 2, it is evident that the bending deformation was larger in the direction perpendicular to the impact path. As shown in Figure 9c, for the laser peening path inclined in the horizontal direction (path 3), a torsionally deformed part was obtained due to one-sided constraints on the plate, resulting in a larger superposition of bending moments perpendicular to the impact path. As shown in Figure 7c, the bending and twisting parts did not coincide with the flat plate at one vertex, with the measured distance being 2.9 mm.

#### 3.1.2. Effect of Different Laser Energies on the Bending Deformation of Aluminum Alloy Plate

**Figure 10:**
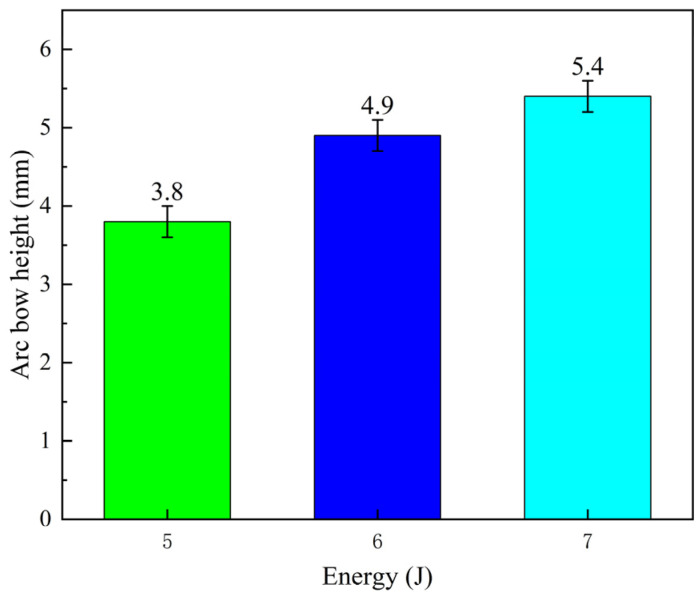
Effect of different laser energies on the bending deformation of aluminum alloy plate.

**Figure 11:**
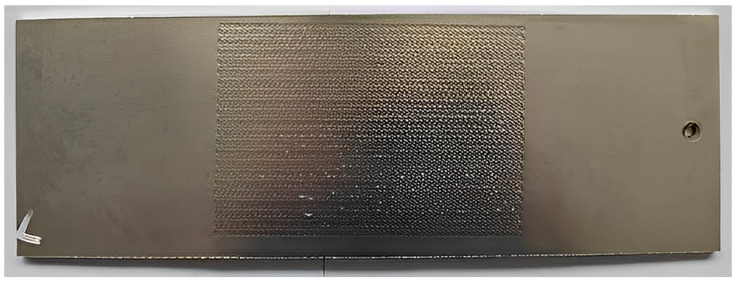
Aluminum alloy plate forming effect under laser energy 7 J.

The effect of different laser energies on the amount of bending deformation of the aluminum alloy plate is shown in Figure 10. As the laser energy increases, the bending deformation also increases. According to Fabbro’s research [21], this is primarily due to the increase in shock-wave force and residual stress within the material, which strengthens the bending moment perpendicular to the laser peening path. The bending moments in other directions are limited or canceled out, leading to increased bending deformation perpendicular to the impact path as laser energy increases. Unlike the effect of the laser peening path on the amount of deformation, the impact path has a more significant influence on the amount of deformation. After laser peening, the black tape on the surface was removed, and the forming effect with 7 J laser energy is shown in Figure 11. On the surface, some micro-pits can be observed, indicating the impact of laser peening forming.

#### 3.1.3. Effect of Thickness on Laser Peening Forming of Aluminum Alloy Plates

**Figure 12:**
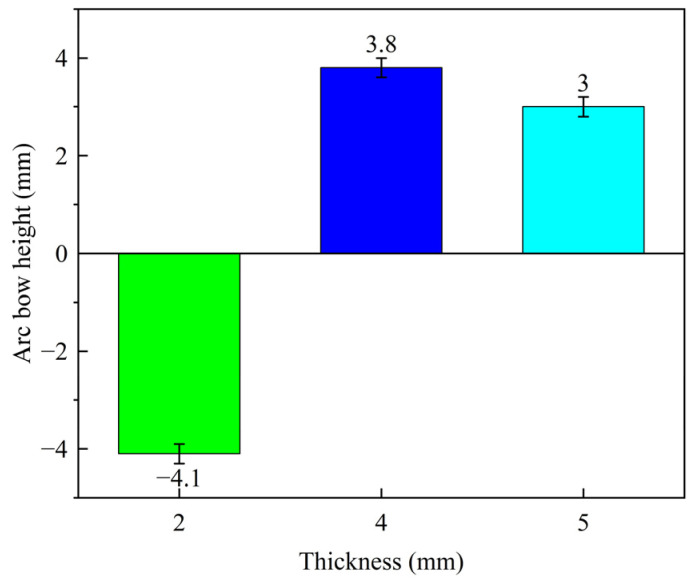
Effect of thickness on laser peening deformation.

**Figure 13:**
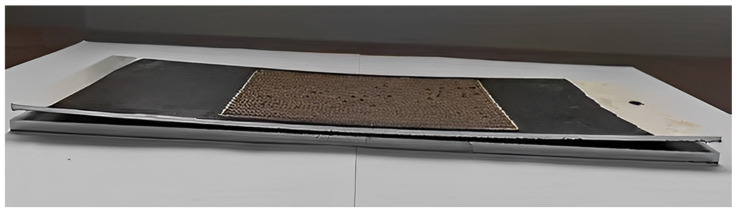
Effect of laser peening forming of aluminum alloy plate with a thickness of 2 mm.

The effect of different thicknesses on the laser peening deformation of aluminum alloy plates is shown in Figure 12. Under the same laser process parameters, the arc bow height is 4.1 mm, 3.8 mm, and 3 mm for plate thicknesses of 2 mm, 4 mm, and 5 mm, respectively. The value of the arc height decreases as the thickness of the plate increases. For the 2 mm-thick aluminum alloy plate, concave deformation occurs relative to the direction of laser incidence, as shown in Figure 13. According to Yang et al. [22], the deformation pattern of the plates is consistent. This is mainly due to the fact that when the aluminum alloy plate is thin, a certain amount of laser energy results in minimal attenuation of the laser-induced shock wave through the entire thickness. Consequently, the bottom surface of the aluminum alloy plate also undergoes plastic deformation, generating a positive bending moment that causes concave deformation. When the thickness of the plate is 4 mm and 5 mm, residual stresses are formed in the laser shock area, a steep stress gradient is formed inside the plate due to the limitation of the surrounding material, and the amplitude gradually decreases, leading to the formation of convex deformation.

### 3.2. Effect of the Number of Laser Shocks on the Surface Quality of Aluminum Alloy Materials

#### 3.2.1. Surface Topography of the Material after Treatment with Different Laser Shock Times

**Figure 14:**
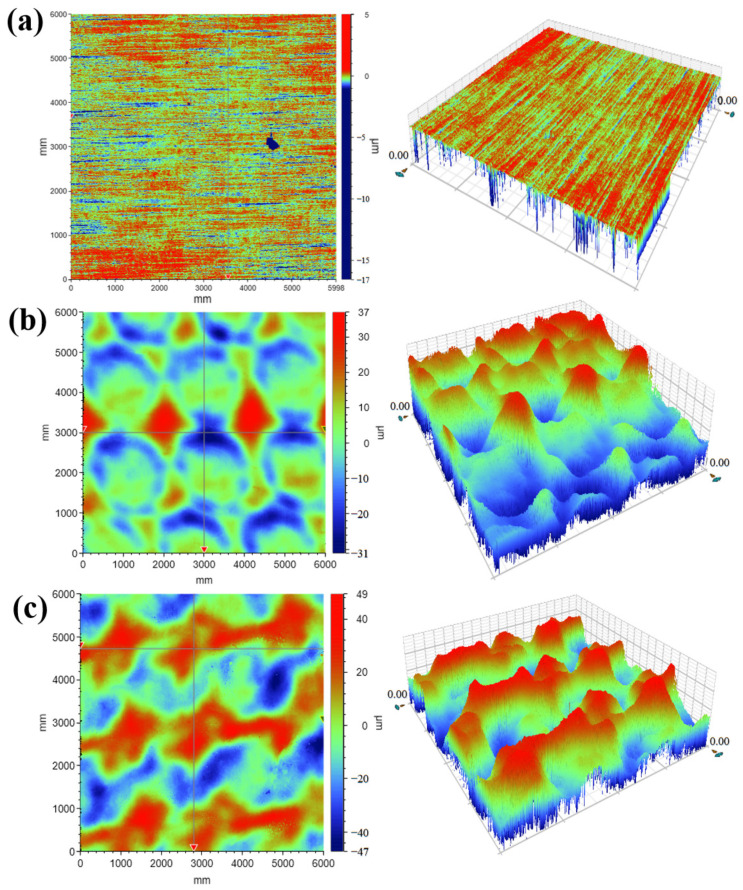
Surface morphology of different specimen pieces: (**a**) Specimen 1; (**b**) Specimen 2; (**c**) Specimen 3.

**Figure 15:**
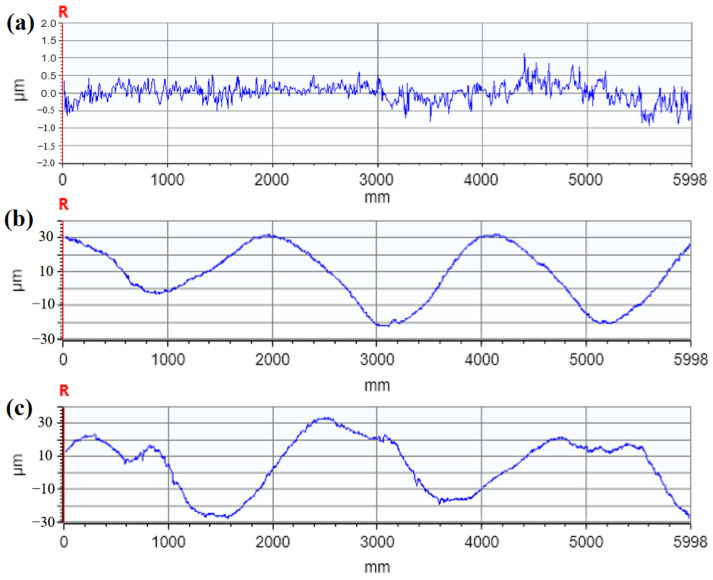
Surface profiles of different specimen pieces: (**a**) Specimen 1; (**b**) Specimen 2; (**c**) Specimen 3.

**Figure 16:**
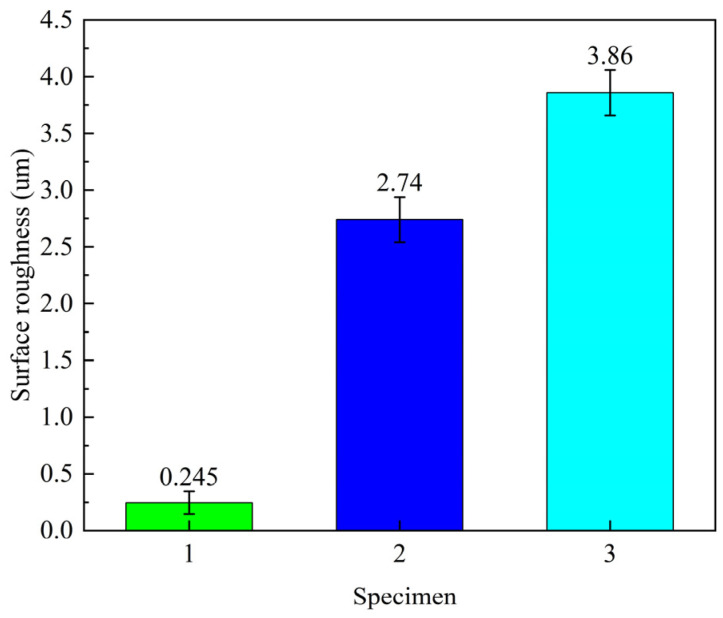
Surface roughness of different specimen pieces.

The surface morphology of different specimens after laser peening is shown in Figure 14, and the contour lines are presented in Figure 15. The surface morphology fluctuations were 22 µm, 68 µm, and 96 µm for specimens 1, 2, and 3, respectively. The surface roughness values were 0.245 µm, 2.74 µm, and 3.86 µm for specimens 1, 2, and 3, respectively, as shown in Figure 16. Figure 15b,c shows the contour plots after 1 or 2 laser shock treatments. The periodicity in the graphs is mainly related to the overlapping ratio, with a concave deformation in the center of the spot and a convex deformation in the rest of the area. It can be seen that with the increase in the number of laser shock treatments, the surface morphology fluctuation of the specimen increases, and the surface roughness increases, which is consistent with the pattern obtained by Qiao et al. [23]. In their study, roughness increased with the increase in the number of laser shock peening; the roughness of the specimen increased from 0.05 μm to 0.37 μm after laser shock peening with 9 J laser pulse energy three times. The increase in surface roughness and morphology fluctuation after laser peening is primarily due to the plastic deformation of the material surface caused by the shock-wave pressure exceeding the material’s dynamic yield strength.

#### 3.2.2. Material Hardness after Different Laser Shock Times

**Figure 17:**
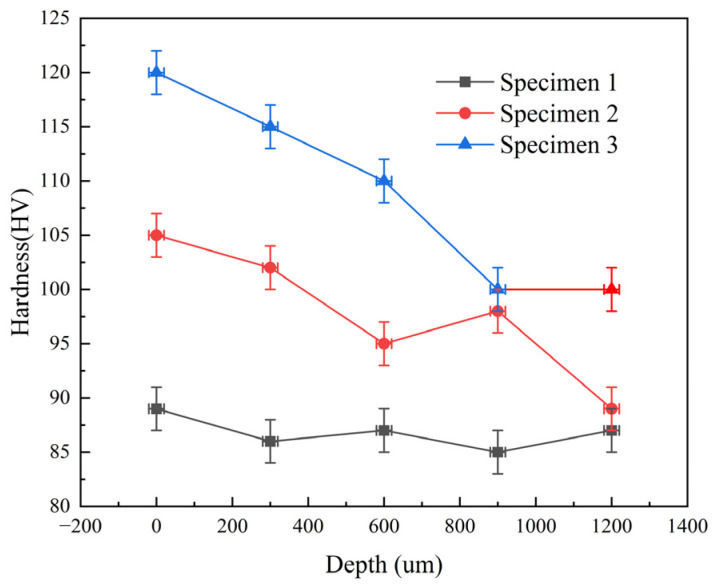
Hardness distribution of different specimen depth directions.

The depth-direction microhardness distribution of the laser-shocked and untreated specimens is shown in Figure 17. The hardness of the untreated 5083 aluminum alloy matrix was 89 HV. After one and two laser double-sided impacts, the hardness increased to 105 HV and 120 HV, respectively, representing increases of 16 HV and 31 HV. In the depth direction, the hardness of the laser-shocked specimens gradually decreased with increasing distance from the surface. Therefore, while laser peening strengthening has a diminishing effect with depth, the impact extends to a depth greater than 1.2 mm. This is due to the fact that a gradual work-hardening layer can easily be formed by the plastic deformation produced under the pressure of the laser shock wave. The intense plastic deformation of the specimen leads to high dislocation density and grain refinement, and the increase of grain boundaries after grain refinement enhances the intergranular bonding [24,25].

#### 3.2.3. Residual Stresses in Materials after Laser Shock Peening

**Figure 18:**
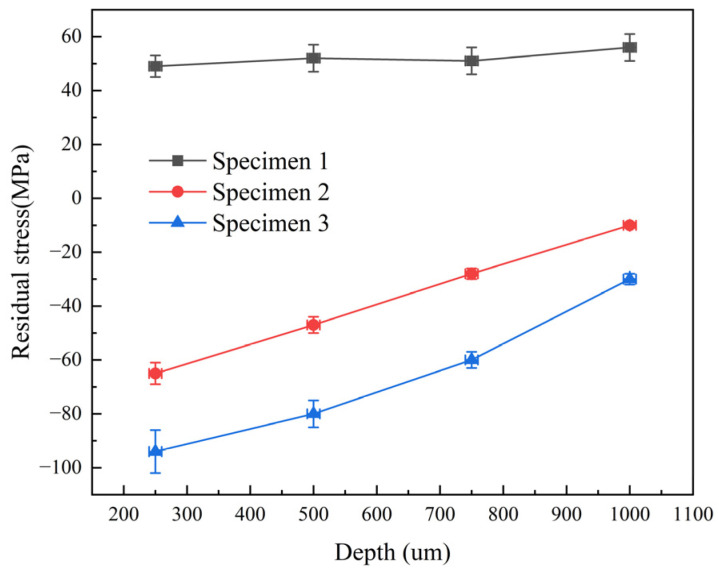
Residual stress distribution in different depth directions of specimens.

The depth-wise distribution of residual stresses in both laser-peened and untreated specimens is depicted in Figure 18. The 5083-aluminum-alloy matrix surface exhibited an initial tensile stress of 58 MPa. Following double-sided laser impacts for one and two cycles, the surface residual stresses shifted to −80 MPa and −107 MPa, respectively, transitioning from tensile to compressive stress states. This transition primarily arose from material plastic deformation induced by laser peening. Plastic deformation was confined to a localized region, counteracted by the surrounding material, resulting in residual compressive stresses. Residual stresses gradually diminished in the depth direction away from the surface in laser-treated specimens [26,27]. Consequently, the reinforcing effect of laser peening attenuated with increasing depth.

### 3.3. Effect of Different Laser Shock Times on the Properties of Aluminum Alloy Materials

#### 3.3.1. Tensile Properties

**Figure 19:**
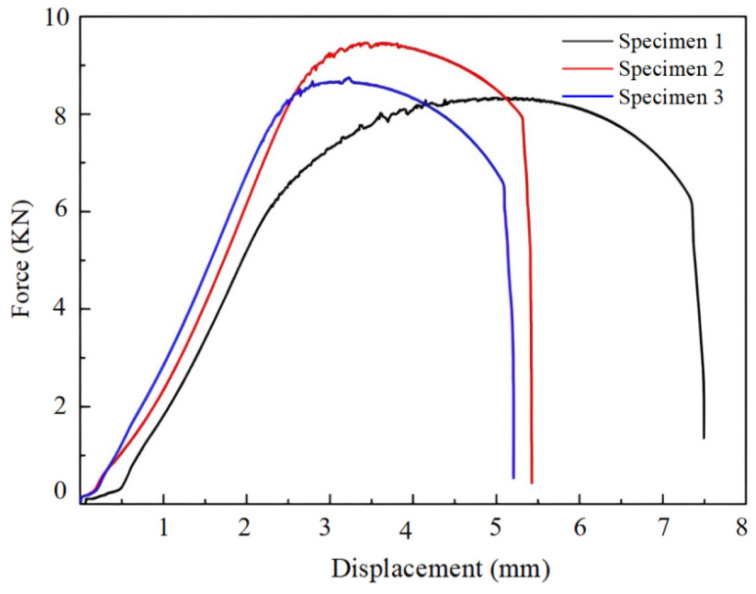
Force-displacement curves for different specimens.

The Force-Displacement curves of the laser-peening-treated specimens are illustrated in Figure 19. Specimens 1, 2, and 3 are the specimens after 1, 2, and 3 laser impacts, respectively. The tensile test results are presented in Table 2. The tensile strengths of specimens 1, 2, and 3 were 208.5 MPa, 218 MPa, and 236.5 MPa, respectively. As can be seen from Table 2, the tensile strength of the specimen increased with the increase in the number of laser shocks. Laser peening treatment demonstrated a significant enhancement in the tensile strength of aluminum alloy materials.

#### 3.3.2. Fracture Morphology

**Figure 20:**
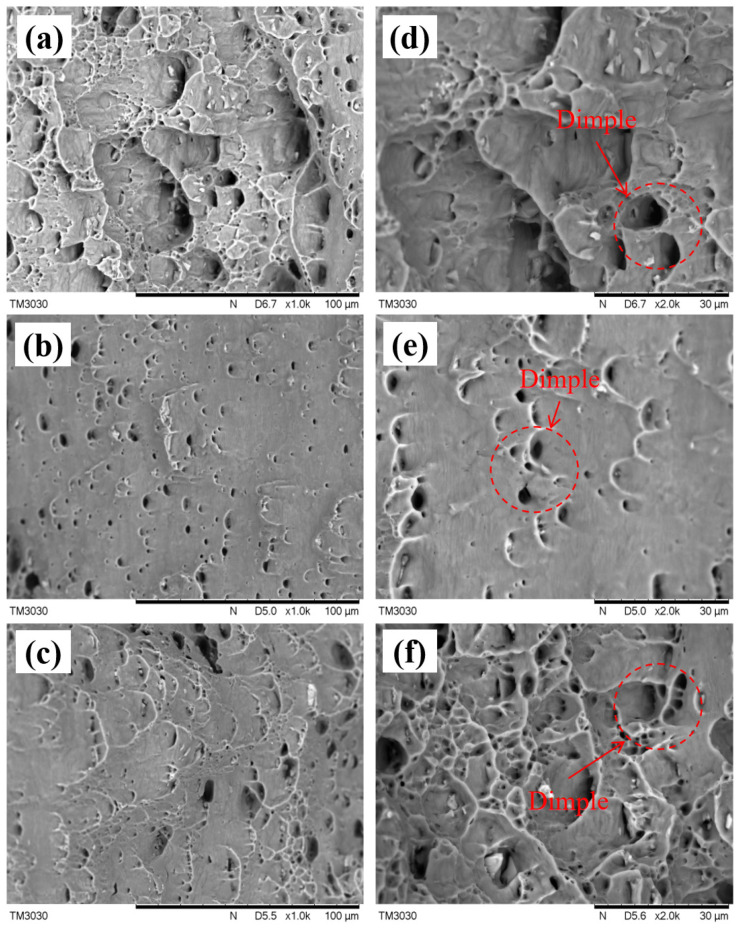
Fracture morphology of 5083 aluminum alloy specimen: (**a**) Specimen 1 (100 um); (**b**) Specimen 2 (100 um); (**c**) Specimen 3 (100 um). (**d**) Specimen 1 (30 um); (**e**) Specimen 2 (30 um); (**f**) Specimen 3 (30 um).

The fracture morphology of the 5083 aluminum alloy specimen is depicted in Figure 20. The tensile fracture exhibited numerous equiaxial dimples, with the dimple in unshocked specimen 1 being larger and unevenly distributed. In contrast, the dimples in the fracture of the laser-shocked specimen were smaller and exhibited a more uniform distribution, indicating refinement post-laser shock. This refinement of the dimple was mainly due to the large number of dislocations during laser shock peening. For specimen 3, the dimples increased and became finer and more pronounced; this is because each impact caused further plastic deformation, and the cumulative effect led to deeper dimples. In addition to this, the dimples of specimen 3 were more evenly distributed on the material surface.

## 4. Conclusions

Firstly, the effects of the laser peening path, laser energy, and plate thickness on the deformation trend and deformation amount of 5083 aluminum alloy plates were investigated. Secondly, the effects of different impact times on the surface quality and tensile properties of 5083 aluminum alloy were examined. In addition, laser peening forming experiments were conducted on aluminum alloy substrates of different shapes. Finally, the effects of various laser peening durations on the surface morphology and tensile properties of the plates were analyzed. The main conclusions are as follows:

(1) The bending moment perpendicular to the direction of the laser peening path is substantial, with the bending deformation reaching up to 12.5 mm. When the laser peening path is inclined horizontally, the aluminum alloy plate is more susceptible to torsional deformation.
(2) Under laser energy conditions of 5 J, 6 J, and 7 J, the deformations were 3.8 mm, 4.9 mm, and 5.4 mm, respectively. As the laser energy increased, the deformation of the plate also increased, with a maximum deformation of 5.4 mm.
(3) For a 4 mm-thick aluminum alloy plate, convex deformation occurred in the direction of laser incidence, and the deformation magnitude increased with higher laser energy. Compared to the impact of laser energy on bending deformation, the impact path had a more significant effect. In the case of a 2 mm-thick aluminum alloy plate, concave bending deformation was observed relative to the laser incidence direction.
(4) After 0, 1, and 2 laser peenings, the surface morphology fluctuations were 22 µm, 68 µm, and 96 µm, respectively, and the roughness was 0.245 µm, 2.74 µm, and 3.86 µm. The surface hardness of the specimens was 89 HV, 105 HV, and 120 HV, with hardness decreasing along the depth direction. The residual stresses on the sample surfaces were 58 MPa, −80 MPa, and −107 MPa, indicating a change from tensile to compressive stresses, with residual compressive stresses diminishing with depth.
(5) After 0, 1, and 2 laser peenings, the tensile strength increased to 208.5 MPa, 218 MPa, and 236.5 MPa, respectively. The tensile fracture surface exhibited finer and more uniformly distributed dimples, demonstrating enhanced plasticity and strength of the material post-laser peening.

## Figures and Tables

**Table 1:** Laser peening process parameters.

Specimen	Number of Shocks	Laser Energy (J)	Overlap Ratio (%)	Pulse Width (ns)
1	0	0	0	0
2	1	5	30	16
3	2	5	30	16

**Table 2:** Tensile test results.

Specimen	Tensile Strength/MPa	Maximum Force/KN	Maximum Displacement/mm
1	208.5	8.34	7.495
2	218	8.75	5.207
3	235.5	9.46	5.424

## Data Availability

The original contributions presented in the study are included in the article; further inquiries can be directed to the corresponding author.
